# Giant vesical lithiasis, complication of enterocystoplasty: case report

**DOI:** 10.11604/pamj.2018.31.132.15995

**Published:** 2018-10-22

**Authors:** Robert Nang, Hadiya Hinchi, Taoulouth Lafia, Mohamed Rami, Rachid Belkacem

**Affiliations:** 1 Service of Pediatric Urology and Plastic Surgery, Rabat Children’s Hospital, Ibn Sina University Hospital, Mohammed V Faculty of Medicine, Rabat, Morocco

**Keywords:** Enterocystoplasty, giant lithiasis, one port trocar

## Abstract

Frequent and recurrent pathology, bladder stone is a constant complication in enterocystoplasty enlargement. The occurrence of this lithiasis is due to some factors such as urinary tract infections, mucus secretion by the intestine segment moved, poor urinary emptying and foreign bodies (sutures, staples) intra-vesical. Clinical signs are not specific. However the formation of giant lithiases remains exceptional. Some cases have been documented in the literature. The occurrence of this affection must be prevented by dietary measures and regular follow up. New therapies for the control of mucus in the bladder tank have been emerged. Open surgical remains the most common. Today, One port trocar endoscopy enables to handle and extracted lithiases of every size.

## Introduction

The occurrence of lithiasis is common in bladder enlargement by a segment of the digestive tract. The occurrence of theses lithiases rely on the recrudescence of supporting factors such as: urinary tract infections, mucus secretions, occurence of an intra vesical foreign body. Clinical observations are a combination of urinary symptoms like: hematuria, pollakiuria, micturition burns and a feeling of heaviness under the umbilicus. The occurrence of giant lithiasis is rare with only 07 cases documented in the literature. These are lithiases of more than 5 cm length and more than 50 grams weight. Our study is reporting the case of a giant intra-vesical lithiasis occurred in a girl under care for bladder exstrophy.

## Patient and observation

**Figure 1:**
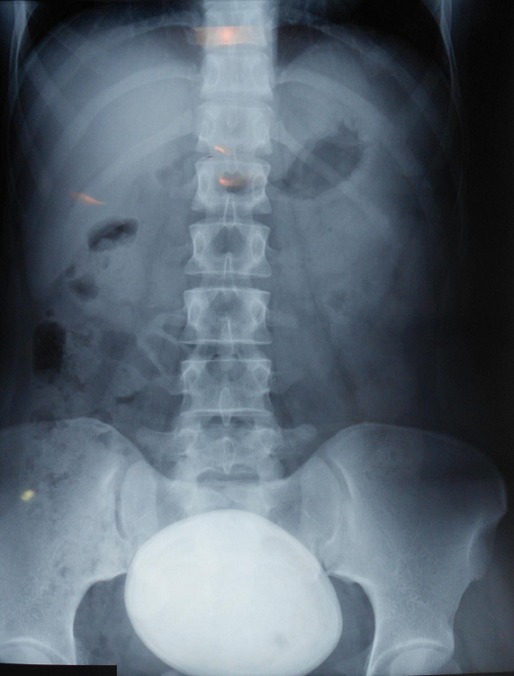
X-ray of urinary tract, showing a calcic opacity in the entire bladder

**Figure 2:**
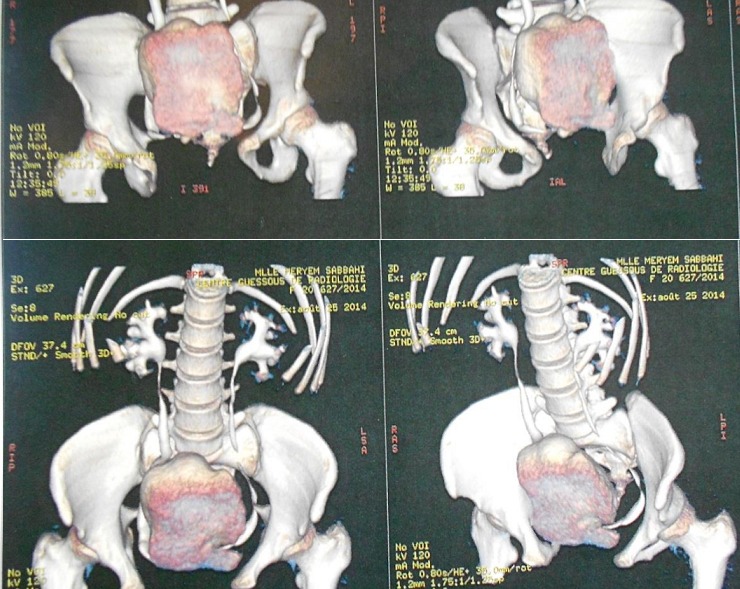
Uroscanner showing giant lithiasis occupying the bladder

**Figure 3:**
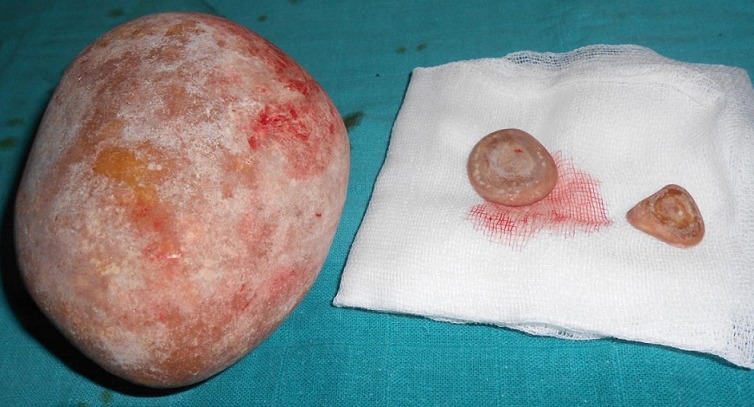
Giant lithiasis and two small stone after their extraction from the neo bladder

It was 21 years old patient under care for bladder exstrophy, experiency a closure of the bladder plate at 2 months without any relapse. She had repetitive urinary infections more or less symptomatic, with day and night urinary leakage. At the age of 7, she was readmitted for enterocystoplasty enlargement with plasty of the bladder neck. At 9 years old, following the persistence of urinary leakage, another surgery took place in order to achieve a trans-appendicular cystostomy of mitrofanoff type. Supported later by an anti-cholinergic drugs protocol, but she did not follow-up. The history of the current illness back to 06 months through the occurrence of a belly overloads feeling without other associated sign. The evolution was marked by total hematuria and urinary leakage. The clinical examination showed a good shape with, a 37°C body temperature, normally colored conjunctiva, a medial umbilical scar, the presence of the permeable and functional intermittent sounding hole to the right iliac fossa, and a mass of firm consistency, mobile and non painful under umbilical palpation. At the X-ray of urinary tract, there was a calcic opacity that invaded the entire bladder (Figure 1). On the scanner among the three intravesical stones were a giant lithiasis (Figure 2). Nothing wrong was found through urine cytobacteriological examination. All the kidney function was good. Surgery was about the resumption of the formal abdominal incision under the umbilical median. The exploration show three lithiases, one giant of 8 cm length and 935g weight and two minor ones (Figure 3). Lithiasis extraction, the neo bladder wall was normal. The follow up after surgery was very easy leading the patient to leave hospital after 5 days. There were no relapses after three years.

## Discussion

Most of the times enterocystoplasty goes with bladder stone complications. The meta analysis conducted by Matheora *et al.* in 2000 showed a prevalence that varies from 18.2% to 52.5% depending on series, with a decline of 4 years [1]. The average time of occurrence of bladder stone after surgery is 38 months [1]. Lithogenesis combined with mitrofanoff external continent diversion in bladder enlargement is related to three specific risk factors: urinary stasis, urinary tract infection, mucus secretion due to the removal of an intestine segment [2]. Poor compliance with intermittent sounding indicates incomplete bladder emptying leading to urinary stasis. The lasting of urine in the bladder for several hours lead to the growth of crystals such as uric acid dihydrates which may aggregate and launch a local lithiasis process. The role of urinary tract infection with ureolytic organisms is fundamental in the genesis of pediatric lithiasis [2, 3]. Urease is part of some germs such as proteus, klebsiella and pseudomonas. It can translate urea into a matrix protein serving as a mold on which salt minerals will aggregate leading to lithiases. In 80% of cases, proteus is the main germ [4]. The mechanism of this lithiasis is the release of ammonium and carbon dioxide. Through the action of urease on urinary urea, this raises the pH and decreases the solubility of calcium phosphates while increasing the concentration of ammonium. Ammonium combines with phosphate and magnesium, results in the occurence of struvite stones (ammonia magnesium phosphate). Calcium phosphate is the first salt to crystallize in urine when the pH is increased above a value of 6.2. Magnesium phosphate only crystallizes when the pH is above 7 [4, 5]. The removed intestine fragment secretes abundant mucus, especially in the first two years following enterocystoplasty [1, 5]. This mucus will serve as a protein matrix for the nucleation process. Mucus can promote the occurrence of stones directly by acting as an heterogeneous nucleating agent or indirectly by facilitating bacterial growth [4, 6]. Hensle *et al*. have shown in a longitudinal series that irrigation schemes using saline to remove mucus decreased the incidence of bladder stones by 43 to 7% [3, 6].

Another risk factor for lithiasis occurrence is the presence of foreign bodies such as staples, residual wires in the bladder. These foreign bodies increase the occurrence of stones in 13% to 43% cases [6]. Clinical examination may reveal microscopic or macroscopic hematuria by mechanical irritation of the bladder tract [5]. This hematuria may be unique or recurrent, isolated or associated with pain or signs of urinary infection. The management of lithiases in bladder enlargements is based on hygiene and dietary measures and the prevention of risk factors. Maintain a normal diuresis that is 1cc/kg. In case of intermittent sounding, practice 6 to 8 emptyings per day to avoid urinary stasis. Rehydration distributed throughout the day. Although a nocturia can be experienced as troublesome by the patient, waking up at least once in the night followed by immediate rehydration covers the whole of the nycthemeron [5, 7] A biannual medical examination is recommended combine with a radiological, kidney examinations and urine sterilization. Drug treatment for mucus secretion management in the bladder is useful in the prevention of lithiasis occurrence [3, 6]. Fan *et al.* demonstrated that Mitomycin C clearly reduced the concentration of mucus and sialic acid in the urine, without visible lesion of the mucous membrane of the neo- bladder ileum. Therefore, Mitomicin C may provide a new therapeutic approach for the management of mucus secretion in bladder enlargements [6]. According to Khorrami Mh *et al.*, somatostatin and the analog (Sandostatin) resulted in decreased mucus production by the intestinal segment of the neo bladder. Patients receiving Sandostatin no longer require routine postoperative irrigation of the bladder, thus contributing to a shorter hospital stay [7]. N-Acetyl-cysteine introduced into the urine decreases the viscosity of the mucus of the neo-bladder, which is confirmed by the in vivo experiment of Schrier *et al.* [8]. The treatment rely on the morphology and size of the lithiasis, involves open surgery or endoscopic surgery by calculus fragmentation [9]. Open surgery is the most commonly performed and allows to check the integrity of the neo bladder [9]. The percutaneous endoscopic method is the intervention which involves the establishment of a one-port trocar by a short incision with regard to neo-bladder. The lithiasis is placed in an endobag to be fragmented by Lithoclast. The fragments are evacuated by the one-port. Drainage is maintained for a few days before resumption of self-surveys. This technique allows the treatment of lithiasic complications on enterocystplasty by minimizing the approach. The use of endobag allows fragmentation of large lithiasis without risk of dissemination. In case of recurrence, it allows the reuse of the same incision. The one-port trocar allows the manipulation of lithiasis and endobag without the need of a new approach. This technique has many interests in the long-term follow-up of bladder reconstructions. It is perfectly reproducible and responds to all situations regardless of the number or the size of lithiasis [9, 10].

## Conclusion

The occurrence of lithiases in enterocystoplasty is an interesting issue that needs to be investigated. The control of risk factors leading to the occurence of these lithiases in neo-bladder is a beneficial approach enabling to improve the quality of life of these patients Open surgery enable us to extract these stones and check out bladder integrity. The percutaneous endoscopic method with a One-port trocar is less deleterious and suggested even in case of major lithiasis.

## Competing interests

The authors declare no competing interests.
